# Factors Associated With Visual Field Testing Reliability in Children With Glaucoma or Suspected Glaucoma

**DOI:** 10.1016/j.ajo.2024.04.005

**Published:** 2024-04-16

**Authors:** ANIKA KUMAR, NATAN HEKMATJAH, YINXI YU, YING HAN, GUI-SHUANG YING, JULIUS T. OATTS

**Affiliations:** University of California, San Francisco School of Medicine, San Francisco, California, USA; Scheie Eye Institute, Center for Preventive Ophthalmology and Biostatistics, Perelman School of Medicine at the University of Pennsylvania, Philadelphia, Pennsylvania, USA; Department of Ophthalmology, University of California, San Francisco, California, USA

## Abstract

**PURPOSE:**

To evaluate Humphrey Visual Field (HVF) test reliability and its associated risk factors in children with glaucoma or glaucoma suspect.

**DESIGN:**

Retrospective cohort study.

**METHODS:**

None.

**SETTING:**

Single-center childhood glaucoma clinic.

**PATIENT POPULATION:**

One hundred thirty-six patients aged ≤18 years with glaucoma/glaucoma suspect, and least 1 completed 24 to 2 HVF test between 2018 and 2023.

**OBSERVATION PROCEDURE:**

Demographic and clinical characteristics including age, primary language, visual acuity (VA), and glaucoma diagnosis were extracted from electronic health records.

**MAIN OUTCOME MEASURES:**

HVF 24 to 2 testing metrics, including FP, FN, and FL. Tests were defined as reliable using manufacturer guidelines of ≤33% FP, ≤33% FN, and ≤20% FL. For each patient, a reliability score was calculated as the percentage of reliable tests among all tests completed. A multivariable logistic regression model was used to determine factors associated with test-level reliability (yes/no). A multivariable linear regression model was used to determine factors associated with patient-level reliability score.

**RESULTS:**

Among 634 HVFs from 136 patients (Mean ± SD age at first test 12.0 ± 3.2 years, 47.8% female), 51.3% were reliable. Older age, better baseline VA, and English as primary language were associated with greater odds of test-level reliability (*P* < .04). Mean ± SD patient-level reliability score was 51.7 ± 38.1%. Older age at first clinic visit, better baseline VA, and English as primary language were associated with higher reliability scores (all *P* < .02), and number of prior VF tests was not (*P* = .56).

**CONCLUSIONS:**

Younger age, worse visual acuity, and non-English as primary language were associated with decreased reliability and should be considered when interpreting VF testing in children. A significant learning effect was not observed with repeated testing.

## INTRODUCTION

STANDARD AUTOMATED PERIMETRY (SAP) IS AN essential tool in diagnosing and monitoring glaucoma by providing quantitative and comparative characterization of visual field defects over time.^1^ In adults with glaucoma, regular SAP testing is considered standard of care and the American Academy of Ophthalmology (AAO) recommends testing at least annually,^2^ though some studies have suggested the utility of more frequent testing.^3^ However, in children, SAP is less widely used given concerns about its feasibility and utility. Specifically, the AAO cautions that quantitative visual field testing in children may be limited by poor reliability.^4^ Successful completion of a quantitative visual field test requires a high degree of attention, the ability to understand and follow directions, maintenance of fixation on a central target, and relative stability of head position during the test: all of which can be challenging for children.^5–7^

Humphrey Visual Field (HVF) testing (Humphrey Instruments, Inc., San Leandro, CA) is one of the most commonly used types of SAP. In HVF testing, reliability is determined by three key metrics: false positives (errors where the test-taker responds when no stimulus is shown), false negatives (errors where the test-taker does not respond to a stimulus of higher intensity where they have previously responded to a lower intensity stimulus), and fixation losses (errors where the test-taker responds to a stimulus within their blind spot, an indication of inconsistent fixation on a central target).^8^ While studies have characterized reliability in visual field testing in adults,^1,9,10^ few have evaluated reliability metrics in children. The goal of this study was to characterize HVF test reliability and its longitudinal change over time in a cohort of children with glaucoma or glaucoma suspect, and to evaluate the factors associated with HVF reliability. Understanding patterns and factors affecting reliability in children can help inform visual field-testing strategy and interpretation.

## METHODS

### STUDY POPULATION AND DATA COLLECTION:

This study was approved by the Institutional Review Board of the University of California, San Francisco and was conducted in adherence with the tenets of the Declaration of Helsinki. This was a retrospective cohort study of children ≤18 years of age seen in a childhood glaucoma clinic between August 2018 and June 2023. Patients were included if they had a diagnosis of glaucoma or glaucoma suspect as defined by the Childhood Glaucoma Research Network classification system,^11^ and at least 1 HVF test (24–2 Swedish Interactive Threshold Algorithm (SITA) Standard or SITA Fast). Eyes with a best-corrected visual acuity (BCVA) of counting fingers or worse were excluded. Demographic data were abstracted from the chart including age, sex, primary language, insurance type, and self-reported race and ethnicity. For patients under the age of 18, primary language recorded in the electronic health record reflected primary language of the patient’s parents. Clinical data collection included glaucoma diagnosis, BCVA in logMAR units, and number of visual field tests completed per eye. For each visual field test, date, test strategy, visual field index (VFI), mean deviation (MD), and test duration were extracted. Reliability metrics were collected including percentage of false positives (FP), percentage of false negatives (FN), and percentage of fixation losses (FL).

### VISUAL FIELD RELIABILITY:

Each test was categorized as unreliable or reliable based on meeting criteria for all three reliability indices per manufacturer guidelines: ≤33% false positives, ≤33% false negatives, and ≤20% fixation losses.^12^ Additionally, for each patient, a reliability score was calculated as the proportion of reliable tests among all tests performed during the study period.

### STATISTICAL ANALYSIS:

Univariate linear regression analyses were performed to determine factors associated with each of the three reliability indices (FP, FN, and FL) on the test-level. Using the significant variables from the univariate analyses, three multivariable mixed effects models (one each for FP, FN, and FL) were performed that included factors that were significant in the univariable analyses (eg, age, primary language, glaucoma diagnosis, BCVA, HVF testing strategy). These models accounted for both correlations between two eyes from the same child and correlations from multiple longitudinal tests from the same eye. A subsequent multivariable repeated measures logistic regression model was performed to investigate the factors associated with binary test reliability (eg, reliable yes/no). Finally, a multivariable linear regression model was performed to investigate the relationship between patient-level reliability score by considering age at first test, sex, primary language, BCVA of the better-seeing eye, glaucoma diagnosis, and testing experience (defined as the total number of tests completed during the study period).

Among eyes with 2 or more completed visual field tests, we evaluated the stability of visual field test results over time by comparing the first and the last tests for VFI, MD, and reliability metrics, and calculating their Pearson correlation coefficients and mean differences. In addition, eyes were grouped into three reliability groups: stable (first and final tests were either both unreliable or both reliable), improved (first test was unreliable but final test was reliable), or worsened (first test was reliable but final test was unreliable). One-way analysis of variance (ANOVA) for repeated measures were used to evaluate demographic and clinical differences across these three VF reliability groups.

All statistical analyses were performed in JMP Pro (17.0) and R (Version 4.1.0, R Foundation for Statistical Computing, Vienna, Austria. https://www.R-project.org/). Two-sided *P*-value <.05 were considered statistically significant. The Strengthening the Reporting of Observational Studies in Epidemiology (STROBE) reporting guideline was followed.

## RESULTS

**FIGURE 1.**
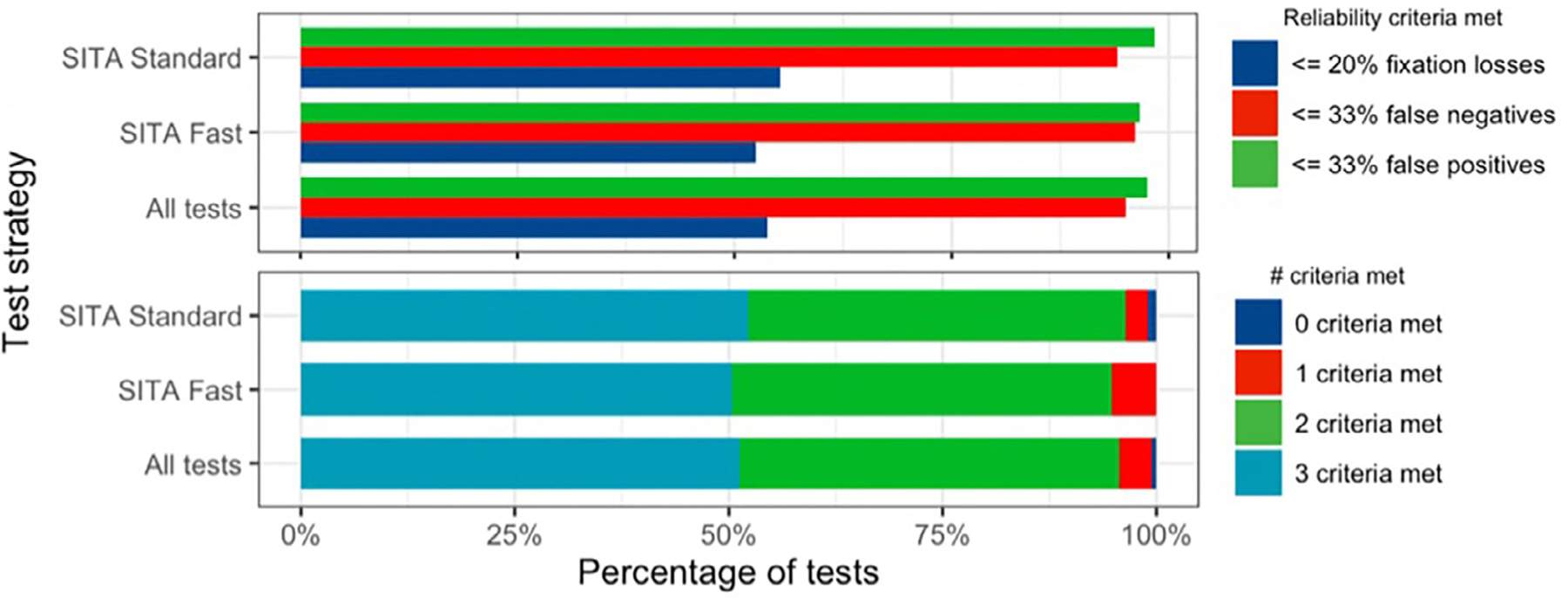
Percentage of tests meeting reliability criteria.

A total of 634 HVF tests from 247 eyes of 136 patients were included in the analysis (Table 1). Mean age ± standard deviation (SD) at first visual field test was 12.0 ± 3.2 years (range: 5–18 years) and 47.8% were female. Sixty-three patients (46.3%) had glaucoma in at least 1 eye and the remaining 73 (53.7%) were glaucoma suspects. The majority of patients (76.5%) spoke English as their primary language. Of the 634 HVF tests included, 304 (47.9%) were 24 to 2 SITA Standard and 330 (52.1%) were 24 to 2 SITA Fast (eTable 1). Mean number of visual field tests completed per patient was 2.74 ± 1.99 tests (range 1–11). Among 300 tests from 105 eyes with glaucoma, mean MD was −9.37 ± 9.47 dB and mean VFI was 79.8% ± 24.0%. Among 314 tests from 131 eyes with glaucoma suspect, mean MD was −3.47 ± 4.96 dB and mean VFI was 92.8% ± 15.0%. For individual reliability metrics, 618 (97.5%) tests were reliable based on FP, 603 (95.1%) based on FN, and 341 (53.8%) based on FL. Only 325 (51.3%) tests were reliable based on criteria for all three reliability metrics (Figure 1).

In multivariable mixed effects models for each VF reliability metrics, non-English as a primary language was associated with a 1.19% increase in false positives (*P* = .008, eTable 2). Worse baseline BCVA was associated with greater false negatives, with a 0.6% increase in false negatives per line of visual acuity (0.1 logMAR, *P* < .0001, eTable 3). Younger age and worse baseline BCVA were both associated with greater fixation losses, with a 2.68% increase in fixation losses for every year decrease in age (*P* = .005) and a 3.48% increase per line worsening of visual acuity (*P* < .001, eTable 4).

In the multivariable repeated measures logistic regression model, older age, better BCVA at the time of VF test, and English as primary language were associated with greater odds of test-level reliability, as defined as meeting all three reliability criteria (adjusted odds ratio [aOR] = 1.27 per year increase in age, 1.32 per 0.1 line of visual acuity better, and 2.14 for English; all *P* < .04, Table 2).

On the patient-level, mean ± SD reliability score was 51.7% ± 38.1%. In the multivariable linear regression model, older age, better BCVA at first visual field test, and English as primary language were all associated with a greater reliability score (all *P* < .023, Table 3). Sex, diagnosis of glaucoma, and number of completed visual field tests were not associated with reliability score (*P* > .27).

Of the 247 eyes included in our study, 164 (66.4%) had data for ≥ 2 visual field tests and were included in the trend analysis to determine if reliability remained stable, improved, or worsened between first and last visual field test. Reliability remained stable in 106 eyes (64.6%), improved in 40 (24.4%), and worsened in 18 (11.0%; Table 4). Mean ± SD time interval between first and last test was 2.29 ± 1.68 years (range, 0.09 to 7.56 years), which was not significantly different among groups (*P* = .22). Average level of experience (measured by number of total visual field tests completed) was 3.37 ± 1.72 tests (range 2–11 tests) and was also similar among groups (*P* = .41). Additionally, mean age at the first test was 12.34 ± 3.24 years (range 5–18 years) and was comparable among groups (*P* = .67). All three reliability indices (FP, FN, and FL) as measured on first and final tests were significantly correlated (Pearson’s correlation coefficient [*r*] = 0.52 for FP, 0.49 for FN, and 0.74 for FL, all *P* < .0001). Mean differences ± SD between first and final test were 1.5 ± 10.1% for FP, −0.2 ± 11.8% for FN, and 5.8 ± 34.6% for FL. VFI and MD values on first and final tests were also correlated (Pearson’s correlation coefficient [*r*] = 0.72 for VFI and 0.75 for MD, all *P* < .0001) with mean differences ± SD between first and final tests of −0.8 ± 5.4 dB for MD and −2.1 ± 17.1% for VFI.

## DISCUSSION

In our study, approximately half of all HVF tests were found to be reliable and fixation losses were the most common cause of unreliable results. Older age, better best-corrected visual acuity, and English as primary language were associated with higher test and patient level reliability. Interestingly, experience with visual field testing, as measured by number of completed visual field tests during 5-year the study period, was not associated with reliability or change in reliability. In patients with repeated visual field testing, reliability metrics at the first and final test were significantly correlated.

Our finding that approximately half of 24 to 2 HVF tests were reliable is similar to previously reports in children. For example, 1 study of 10 children with glaucoma found a reliability rate of 47.5%, though with slightly different reliability criteria (FP, FN, and FL all < 25%).^13^ Despite concerns that reliability is a bigger challenge in visual field testing in children, overall reliability in our study is also similar to several studies in adults. Two studies of adults with and without glaucoma using the same reliability criteria as our study found overall reliability rates between 52% and 59.5%.^1,10^ Like our study, fixation losses were the most common reason for lack of reliability. In contrast, another study of adults with glaucoma found that median percentage of fixation losses was 7%, much lower than our study, though this study only included patients with a BCVA of 20/40 or better, limiting the generalizability of these findings to other populations.^9^

Indeed, we found that better BCVA was associated with increased test reliability and lower rates of fixation losses and false negatives. While 1 study of adults with glaucoma found no significant relationship between BCVA and the reliability indices of FP, FN, or FL,^14^ research examining such associations in children is limited. Our findings indicate potential differences in the effect of BCVA on reliability in children and adults with glaucoma. Additionally, in our study, older age was associated with increased reliability and decreased fixation losses. The effects of age on visual field reliability in children has not been extensively investigated. One study found that children over 10 years of age tended to have fewer abnormal points per visual field test compared to younger children.^13^ These findings provide support to the hypotheses that younger children who may have shorter attention spans, differences in motivation, less refined cognitive and motor skills, and more difficulties fix-ating may be more likely to produce unreliable visual field tests.^15,16^ We also found that non-English as primary language was associated with worse test reliability. This may be related to challenges with understanding VF test instructions, even when an interpreter was used. This finding may have also been related to barriers to parent understanding of test instructions, given that primary language recorded in the health record reflected parent proficiency in this population and that parents were sometimes involved in assisting the test administrator’s communication of instructions to the child, In contrast to our study, 1 Israeli-based study concluded that language was not associated with reliability and that using an interpreter in adult patients’ native languages to administer the HVF test did not affect reliability parameters.^17^ We could find no studies examining this association in children, so our findings may be the first to indicate that children, as compared to adults, may experience less success in the setting of interpreters used during HVF test administration. Altogether, these findings suggest that visual field interpretation may be limited in younger children, those with worse visual acuity, and those whose primary language is not English.

We performed a longitudinal analysis to evaluate whether the reliability of visual field testing changed over time and found that the majority remained stable and only 24.4% improved over a mean of 2.3 years. Though we hypothesized that level of experience or “learning effect” with repeated tests could contribute to reliability, we found that an increased number of completed tests was not associated with patient reliability scores. Additionally, there was no difference in number of tests among patients with stable, improved, and worsened reliability over time. These findings are consistent with prior studies in adults that similarly found a nonsignificant relationship between total number of tests and reliability indices.^9,18^ In contrast, other studies have shown a significant improvement in reliability in adults with and without glaucoma with repeated testing over the course of 1 week to 4 months.^19,20^ It is possible that the contribution of a possible learning effect could have been masked in our analyses by the fact that patients may have been re-referred for VF testing if their prior tests were unreliable; therefore, a greater number of VF tests in some patients may have truly led to a greater ability to perform the test reliably due to a learning effect, but in other patients, may have represented a pattern of unreliability despite any learning effect. Additionally, our finding that most patients belonged to the stable reliability group indicates that changes in reliability are less likely. These findings suggest that providers can expect a relatively high degree of stability in reliability and that children with initially reliable or unreliable tests are likely to perform similarly in future tests.

One limitation of our study is our population’s mean age of 12 years. Given our finding that older age is associated with greater odds of reliability, this may limit generalizability to younger children. Our study also may be limited in its definition of reliability. Though we used HVF manufacturer guidelines to set our reliability criteria, it is possible that measurements of some of the indices may not have been true representations of underlying constructs of reliability.^21^ Specifically, fixation losses are generally a highly variable metric and may be inappropriately increased with changes in head position or an increase in false positives, even if central fixation is maintained.^22^ Additionally, our study is limited in its ability to assess true language proficiency of the patients. It is possible that individuals classified as having a non-English primary language due to parent language preference may actually have been proficient in English themselves. This phenomenon may have biased our study towards underestimating the true association between non-English as a primary language and test reliability. Our analysis also did not account for other factors related to test administration, such as technician performance, which has been demonstrated to significantly affect perimetry results through affecting patient instruction, reassurance during the test, and patient monitoring during the test.^23^ Finally, this analysis was limited to patients with glaucoma and glaucoma suspect, so future investigation will be required to examine reliability trends in children with healthy afferent visual systems.

## CONCLUSION

In a population of children with glaucoma and glaucoma suspect, approximately half of visual field tests were reliable. Adult reliability metrics may need to be modified for childhood visual field testing. Younger age, worse visual acuity, and non-English as primary language were associated with decreased reliability and should be considered when interpreting VF testing in children. A significant learning effect was not observed with testing, and reliability of first test generally predicted reliability of final test.

## Supplementary Material

Supplementary

## Figures and Tables

**TABLE 1.** Demographic and Clinical Characteristics of Included Patients

Characteristic	(*N* = 136)
**Age at first visit, mean (SD) years**	12.01 (3.19)
0–8 years	20 (14.7%)
9–13 years	68 (50.0%)
14–18 years	48 (35.3%)
**Glaucoma diagnosis in eye with worst glaucoma diagnosis, *n* (%)**	
Glaucoma	63 (46.3%)
Suspect	73 (53.7%)
**Type of glaucoma among glaucomatous patients, n (%)**	
Acquired	10 (7.4%)
Associated with nonacquired syndrome/ocular anomaly	12 (8.8%)
Following cataract surgery	12 (8.8%)
Juvenile open-angle glaucoma	6 (4.4%)
Primary congenital glaucoma	23 (16.9%)
**Sex, n (%)**	
Female	65 (47.8%)
Male	71 (52.2%)
**Language, n (%)**	
Cantonese	4 (2.9%)
English	104 (76.5%)
Spanish	25 (18.4%)
Other	3 (2.2%)
**Self-reported race, n (%)**	
Asian	28 (20.6%)
Black or African American	13 (9.6%)
White	24 (17.6%)
Other	53 (39.0%)
Unknown/not reported	18 (13.2%)
**Self-reported ethnicity, n (%)**	
Hispanic/Latino	50 (36.8%)
Not Hispanic/Latino	72 (52.9%)
Unknown/not reported	14 (10.3%)
**Insurance type, n (%)**	
Private	53 (39.0%)
Public	79 (58.1%)
None	4 (2.9%)
**Baseline best-corrected visual acuity, logMAR, mean (SD)**	
Better-seeing eye	0.18 (0.33)
Worse-seeing eye	0.38 (0.47)

**TABLE 2.** Multivariable Analysis for Factors Associated With Humphrey Visual Field Test Reliability

Characteristic	Adjusted Odds Ratio (95%CI)^a^	*P-*Value
Age at time of test, per year increase	1.27 (1.16, 1.39)	<.0001^†^
Primary language		
English	Reference	–
Non-English	0.47 (0.23, 0.95)	.04^†^
Diagnosis of eye		
None	Reference	–
Suspect	0.52 (0.14, 1.99)	.34
Glaucoma	0.89 (0.23, 4.40)	.87
Sex		
Male	Reference	–
Female	0.65 (0.35, 1.19)	.16
Baseline BCVA, per 0.1 logMAR increase	0.76 (0.69, 0.83)	<.0001^†^
Test strategy		
SITA Fast	Reference	
SITA Standard	0.74 (0.44, 1.25)	.26

BCVA = best corrected visual acuity.

a The odds ratio of binary test reliability measured on each visual field test was modeled in a repeated measures logistic regression model.

† p < 0.05

**TABLE 3.** Multivariable Analysis for Factors Associated With Patient Reliability Score

Characteristic	Adjusted Mean Reliability Score^b^	Regression Coefficient (95%CI)^a^	*P-*Value
Age at first visual field test, per year increase		2.60 (0.68, 4.51)	.008^†^
Primary language			
English	55.37%	Reference	–
Non-English	39.59%	−15.78 (−22.56, −9.00)	.02^†^
Diagnosis of eye^c^			
Glaucoma (in at least 1 eye)	46.96%	Reference	–
Suspect (in at least 1 eye)	48.00%	1.04 (−4.83, 7.45)	.87
Sex			
Male	50.75%	Reference	–
Female	44.23%	−6.54 (−12.66, −0.96)	.27
Baseline BCVA of better-seeing eye, per 0.1 logMAR increase		−4.79 (−6.65, −2.93)	<.0001^†^
Number of completed visual field tests		0.86 (−2.11, 3.84)	.57

BCVA = best corrected visual acuity.

a The reliability score was modeled in a multivariable linear regression model. The coefficient indicates the change in average reliability score in percentage points, with positive numbers indicating a higher percentage of tests that were reliable.

b Adjusted mean reliability score indicates the average adjusted mean reliability score for each level of each categorical variable included in the model, adjusted for all other variables included in the model.

c Diagnosis of eye refers to the diagnosis of the eye with the worst glaucoma diagnosis at the initial visual field test.

† p < 0.05

**TABLE 4.** Reliability Group Analysis for Factors for Patients With ≥ 2 HVF Tests

Characteristic	Stable Group	Improved Group	Worsened Group	*P*-Value
Number of eyes	106	40	16	
Time interval between first and final test, mean (SD) years	2.17 (1.65)	2.57 (1.82)	2.38 (1.57)	.22
Number of tests completed, mean (SD)	3.25 (1.80)	3.5 (1.57)	3.78 (1.59)	.41
Age at first test, mean (SD) years	12.42 (3.19)	12.15 (3.49)	12.28 (3.14)	.67
