# Supplementary material for: Factors Associated With Visual Field Testing Reliability in Children With Glaucoma or Suspected Glaucoma
Source: Am J Ophthalmol. Author manuscript; Available in PMC 2024 Aug 1. (PMC11257782; doi:10.1016/j.ajo.2024.04.005)
Supplement: Supplementary [file NIHMS2001308-supplement-Supplementary.docx]

**eTables**

**eTable 1: Humphrey 24-2 Visual Field test characteristics**

| **Characteristic** | **HVF 24-2 Tests**  **(n = 634)** |
| --- | --- |
| **Age at test, mean (SD) years** | 13.19 (3.17) |
| **Eye-Level Diagnosis at time of visual field test**  Acquired  Associated with non-acquired syndrome/ocular anomaly  Following cataract surgery  JOAG  None  PCG  Suspect | 50 (7.9%)  64 (10.1%)  70 (11.0%)  40 (6.3%)  20 (3.2%)  76 (12.0%)  314 (49.5%) |
| **SITA Test Strategy**  Standard  Fast | 304 (47.9%)  330 (52.1%) |
| **Test Parameters, mean (SD)**  False Positives  False Negatives  Fixation Losses  MD  VFI  Duration, seconds | 6.0% (9.6%)  7.2% (11.3%)  35.1% (70.48%)  -6.32 (8.10)  86.5% (21.2%)  305.13 (108.69) |
| **Reliability Criteria**  # tests with < 33% false positives  # tests with < 33% false negatives  # tests with < 20% fixation losses  # tests meeting none of the 3 reliability criteria  # tests meeting 1 of the 3 reliability criteria  # tests meeting 2 of the 3 reliability criteria  # tests meeting 3 of the 3 reliability criteria | 618 (97.5%)  603 (95.1%)  341 (53.8%)  3 (0.5%)  25 (3.9%)  281 (44.3%)  325 (51.3%) |

**eTable 2: Factors associated with percentages of false positives**

| **Characteristic** | **Coefficient (95%CI)^a^** | **p*-*value** |
| --- | --- | --- |
| Age at time of test, pear year increase | -0.17 (-0.43-0.09) | 0.1899 |
| Primary language  English  Non-English | Reference  1.19 (0.32, 2.05) | --  0.0075† |
| Diagnosis of eye  None  Suspect  Glaucoma | Reference  4.15 (-0.21, 8.51)  3.88 (-0.58, 8.33) | --  0.0621  0.0880 |
| Sex  Male  Female | Reference  -0.20 (-0.96, 0.56) | --  0.6063 |
| BCVA, per 0.1 logMAR increase | 0.53 (-1.70, 2.77) | 0.6387 |
| Testing Strategy  SITA Fast  SITA Standard | Reference  -0.71 (-1.52, 0.10) | --  0.0839 |

^a^ The percentage of false positives measured on each visual field test was modeled in a mixed effects multivariable linear regression model. The coefficient indicates the change in the average percentage of false positives, with positive numbers indicating greater false positives.

Abbreviations: BCVA = best corrected visual acuity

**eTable 3: Factors associated with percentages of false negatives**

| **Characteristic** | **Coefficient (95%CI)^a^** | **p*-*value** |
| --- | --- | --- |
| Age at time of test, per year increase | -0.16 (-0.46, 0.14) | 0.3036 |
| Primary language  English  Non-English | Reference  -0.18 (-1.19, 0.84) | --  0.7311 |
| Diagnosis of eye  None  Suspect  Glaucoma | Reference  -3.93 (-9.26, 1.40)  -2.13 (-7.39, 3.12) | --  0.1457  0.4262 |
| Sex  Male  Female | Reference  0.73 (-0.17, 1.62) | --  0.1113 |
| BCVA, per 0.1 logMAR increase | 0.60 (0.34, 0.87) | <0.0001† |
| Testing Strategy  SITA Fast  SITA Standard | Reference  -0.11 (-1.06, 0.84) | --  0.8231 |

^a^ The percentage of false negatives measured on each visual field test was modeled in a mixed effects multivariable linear regression model. The coefficient indicates the change in the average percentage of false negatives, with positive numbers indicating greater false negatives.

Abbreviations: BCVA = best corrected visual acuity

**eTable 4: Factors associated with percentages of fixation losses**

| **Characteristic** | **Coefficient (95%CI)^a^** | **p*-*value** |
| --- | --- | --- |
| Age at time of test, per year increase | -2.68 (-4.56, -0.81) | 0.0050† |
| Primary language  English  Non-English | Reference  -0.98 (-7.27, 5.30) | --  0.7581 |
| Diagnosis of eye  None  Suspect  Glaucoma | Reference  19.82 (-11.79, 51.44)  3.60 (-28.68, 35.89) | --  0.2186  0.8266 |
| Sex  Male  Female | Reference  3.33 (-2.20, 8.86) | --  0.2377 |
| BCVA, per 0.1 logMAR increase | 3.48 (1.86, 5.10) | <0.0001† |
| Testing Strategy  SITA Fast  SITA Standard | Reference  -0.02 (-5.86, 5.82) | --  0.9955 |

^a^ The percentage of fixation losses measured on each visual field test was modeled in a mixed effects multivariable linear regression model. The coefficient indicates the change in the average percentage of fixation losses, with positive numbers indicating greater fixation losses.

Abbreviations: BCVA = best corrected visual acuity
